# *Allium ursinum* and *Allium oschaninii* against *Klebsiella pneumoniae* and *Candida albicans* Mono- and Polymicrobic Biofilms in In Vitro Static and Dynamic Models

**DOI:** 10.3390/microorganisms8030336

**Published:** 2020-02-27

**Authors:** Emilia Galdiero, Valeria Di Onofrio, Angela Maione, Edvige Gambino, Renato Gesuele, Bruno Menale, Martina Ciaravolo, Federica Carraturo, Marco Guida

**Affiliations:** 1 Department of Biology, University of Naples Federico II, via Cinthia, 80100 Naples, Italy; angela.maione3@gmail.com (A.M.); renato.gesuele@unina.it (R.G.); bruno.menale@unina.it (B.M.); federica.carraturo@unina.it (F.C.); marco.guida@unina.it (M.G.); 2 Department of Sciences and Technologies, University of Naples Parthenope, Business District, Block C4, 80143 Naples, Italy; 3 Department of Chemical Sciences, University of Naples Federico II, via Cinthia; 80100 Naples, Italy; martinaciaravolo@gmail.com

**Keywords:** polymicrobial biofilm, CDC biofilm reactor, *Allium* extracts, *Klebsiella pneumoniae*, *Candida albicans*

## Abstract

The present study assesses the in vitro antibiofilm potential activity of extracts of wild *Allium ursinum* and *Allium oschaninii*. The active ingredients of the extracts were obtained with a technique named Naviglio (rapid solid–liquid dynamic extraction, RSLDE) which is based on an innovative and green solid–liquid extraction methodology. The extracts were tested against models of mono- and polymicrobial biofilm structures of clinically antibiotic-resistant pathogens, *Klebsiella pneumoniae* ATCC 10031 and *Candida albicans* ATCC 90028. Biofilms were studied using a static and a dynamic model (microtiter plates and a CDC reactor) on three different surfaces reproducing what happens on implantable medical devices. Antimicrobic activities were determined through minimum inhibitory concentration (MIC), while antibiofilm activity was assessed by minimum biofilm eradication concentration (MBEC) using a crystal violet (CV) biofilm assay and colony forming unit (CFU) counts. Results showed that both *Allium* extracts eradicated biofilms of the tested microorganisms well; biofilms on Teflon were more susceptible to extracts than those on polypropylene and polycarbonate, suggesting that when grown on a complex substrate, biofilms may be more tolerant to antibiotics. Our data provide significant advances on antibiotic susceptibility testing of biofilms grown on biologically relevant materials for future in vitro and in vivo applications.

## 1. Introduction

Biofilms formed by multidrug-resistant bacteria represent a major public health problem because high antibiotic doses are needed to eliminate them. Consequently, toxic effects are commonly reported in patients [1,2].

The spread of antibiotic resistance in the hospital environment and in communities where biofilms are involved has stimulated the development of innovative strategies that can solve this problem. Due to the slow spread of antibiotics through the biofilm matrix, the presence of biofilms represents a challenging eradication issue compared to planktonic bacteria [3].

*Klebsiella pneumoniae* is considered an important opportunistic pathogen in hospitalized patients and can cause extra-intestinal infections, such as urinary tract infections [4], bacteremia [5], device-related infections [6], ventilator-associated pneumonia [7], and community-acquired pneumonia [8].

*K. pneumoniae* can form biofilms, an aggregate in which cells integrated into a self-produced matrix of extracellular polymeric substances adhere to each other and/or to a surface.

Biofilm formation has been identified as one of the major mechanisms of virulence in *K. pneumoniae* human infections.

In addition, *K. pneumoniae* cells within biofilms are partially protected by immune defenses, the matrix blocks access to antibodies and antibacterial peptides, reduces the effectiveness of complement and phagocytosis, and reduces responses that lead to chronic infection.

Recent studies suggest that standard culture techniques do not detect all the bacteria present in the infected joint space, which leads to inadequate antimicrobial therapy [9].

*Candida albicans* is an important fungal pathogen. It is found in more than 90% of human fungal infections and is an important etiological factor in nosocomial infections that involve the formation of a biofilm in medical devices such as urinary catheters, dental prostheses, and silicone implants [10,11,12].

Since no therapy is able to completely eradicate *C. albicans* biofilms, sometimes the removal and/or replacement of the devices is needed [13].

*C. albicans* and *K. pneumoniae* are known to colonize the same habitats [14].

The importance of polymicrobial infections caused by a mixture of bacteria and fungi is increasingly recognized in medical settings and their complexity represents an additional challenge to find an efficient treatment strategy [15].

Antibiotic abuse has led to increased clinical resistance of microorganisms and the occurrence of uncommon infections; many infections are caused by microorganisms that resist response to conventional treatment. It is necessary to develop new antimicrobial agents that can inhibit the formation or destroy mature biofilms, increasing the susceptibility of microbes to antibiotics.

There is a growing interest in the use of compounds derived from medicinal plants as alternative antimicrobial agents.

It has been reported that natural herbal products are a rich source of antimicrobial agents. Garlic (*Allium sativum*) has been considered as a weapon against multidrug-resistant pathogens, it has a broad-spectrum activity against various bacterial, viral, and fungal infections with a low potential for resistance development due to the presence of multiple sites of action [16,17].

Many studies have identified the anticancer, antioxidant, antimicrobial, lipid-lowering, anti-inflammatory, and antiparasitic effects of garlic [18,19,20].

*Allium* is a genus belonging to Amaryllidaceae; it includes many species living in tropical, subtropical, and temperate regions.

The European *Allium ursinum* referred to as “wild garlics” is not well characterized and recent research has evidenced its anticancer, anti-inflammatory, antiviral, antiplatelet, and hypolipidemic effects [21,22]. *A. ursinum* has been used for centuries in traditional medicine and is a perennial herbaceous species, of widespread distribution both in Europe and Asia.

*Allium oschaninii* O. Fedtsch. is a perennial species similar to the common onion, it is spread in the mountainous areas of Central Asia, where it grows wild on rock terraces and stony slopes [23,24].

This study aims to evaluate for the first time the antimicrobial and antibiofilm activities of crude extracts of two *Allium* species obtained with a green method, using a special device the Naviglio extractor that is able to reduce extraction times with a very good yield, and at the same time allows the extraction of the active ingredients avoiding their degradation.

The activity of these extracts from *A. ursinum* and *A. oschaninii* was tested against monomicrobial and polymicrobial biofilms evaluating in parallel the static model of biofilms grown in microtiter plates and a dynamic model of biofilms grown in the CDC reactor.

Considering the great importance of studying biofilms under conditions reproducing as much as possible those founded in the environment of different kind of devices used in the medical field, bacterial and yeast attachment and polymicrobial biofilms formed by *K. pneumoniae* and *C. albicans*, on the outer surface of those materials were compared [21,22,25].

We tested coupons made of polycarbonate, polypropylene, or Teflon because these surfaces are often used for medical devices. Coupon groups were placed in a CDC biofilm reactor (CDC-BR, model CBR 90, Biosurface Technologies Corporation, Bozeman, MT) developed by the Centers for Disease Control and used to study biofilms formed by various types of bacteria. The protocol used by this reactor has been approved by the ASTM (American Society for Testing and Materials) as a standard method for the growth of repeated *Pseudomonas aeruginosa* biofilms on polycarbonate surfaces (designation E 17). The CDC biofilm reactor has been recognized as a reliable and reproducible reactor system to clarify various aspects of physiology, morphology, growth dynamics, and antibiotic sensitivity towards biofilm. The CDC biofilm reactor is a dynamic system in which nutrients are replenished continuously and the capacity can vary from laminar flow to turbulent flow depending on the reactor system. Flow cells are a useful tool to allow detailed studies of the initial attachment and biofilm removal events, two important aspects of biofilm control strategies.

Biofilms grow, in vivo, in a diverse range of conditions, and must therefore be studied in vitro using laboratory dynamic systems that model various conditions and reproduce the fluid flow conditions found under shear stress. This model has the advantage of being inexpensive to establish and easy to reproduce and manipulate [26,27].

We also studied the differences in biofilm formation and antimicrobial tolerance of the two extracts on single species versus on mixed biofilms.

## 2. Materials and Methods

### 2.1. Plant Material and Extraction/Preparation of Garlic Samples

*A. ursinum* and *A. oschaninii* were both obtained from the Botanic Garden of Federico II University.

The garlic extract was obtained with a rapid solid–liquid dynamic extraction (RSLDE) carried out using a Naviglio Extractor^®^ (AtlasFiltri Engineering S.r.l., Padova, Italy). This is an apparatus that allows a rapid solid–liquid extraction, maintaining the liquid in contact with the solid in programmable pressurization–depressurization cycles between atmospheric pressure and 8–9 bar (Naviglio’s Principle) [28].

Garlic (200 g) was gently broken and subsequently extracted using 500 mL methanol as the solvent of extraction. The program was: 2 min for the static phase and 2 min for the dynamic phase; total cycles were 360 and total time of extraction was 24 h. All of the experiments were performed in triplicate.

Main compounds found in the extract contained sulfurated substances [29].

The extract obtained was then filtered into sterile beakers and the filtrate concentrated to dryness by a rotary evaporator at low temperature (30 °C). The dry extract was stored at 4 °C for further use.

The initial concentration of 500 µg/mL was then diluted with sterile distilled water to get concentrations of 5–250 µg/mL, for subsequent uses [30,31].

### 2.2. Strains and Culturing Conditions

Test microorganisms *K. pneumoniae* ATCC 10031 and *C. albicans* ATCC 90028 were maintained in glycerol stock cultures at −80 °C prior to use and propagated by streaking onto tryptone soya agar (TSA) (Oxoid). Single colonies of bacteria and fungi from the overnight cultures were inoculated into tryptone soya broth (TSB) (Oxoid) with 1% of glucose for *C. albicans* and without 1% of glucose for *K. pneumoniae* and then incubated in a shaking incubator at 37 °C.

### 2.3. Antimicrobial Assay

Determination of minimal inhibitory concentration (MIC) of the two extracts was performed by the microdilution method according to Clinical and Laboratory Standards Institute (CLSI) [32], using 96-well microtiter plates and microorganism suspensions prepared in tryptone soya broth with or without 1% glucose. In each well, 100 μL of fresh medium with increasing extract concentrations were introduced and 100 μL of microbial suspension was added, reaching a final concentration of 5 × 10^5^ CFU/mL.

*A. ursinum* and *A. oschaninii* extracts were used at concentrations ranging from 5 to 250 μg/mL.

After 24 h of incubation at 37 °C, absorbance was measured at 590 nm wavelength by the turbidity method using an ELISA plate reader (SINERGY Ha BioTek).

The minimum bactericidal or fungicidal concentration (MBC/MFC) of the two extracts needed to kill ≥99.9% of bacteria was determined by inoculating 10 μL from the wells demonstrating the MIC concentration on TSA incubated for 24 h at 37 °C to count viable cells. The lowest concentration that led to ≥99.9% decrease in CFUs/mL was considered the MBC.

### 2.4. Mono- and Dual-Species Biofilm: In Vitro Static Model

A previously described biofilm assay was performed to assess biofilm formation by examined strains, in 96-well polystyrene plates according to Stepanović et al. [33,34].

Briefly, 200 µL of a cell suspension, containing 1 × 10^7^ cells/mL of each microorganism for monomicrobial biofilms and a mixing of 10^5^ CFU/mL of *C. albicans* and 10^6^ CFU/mL of *K. pneumoniae* for dual-species biofilms prepared in TSB 0.1%, was added to 96-well polystyrene plates. Biofilm formation was allowed to occur for 24 h (to value biofilm formation) or 48 (to value eradication of mature biofilm) at 37 °C. Microorganisms adherent to 96-well polystyrene plates were washed twice with a 0.9% NaCl solution and allowed to dry in an inverted position. The total biomass was measured by crystal violet staining and acetic acid elution as previously described.

The experiments were done in triplicate in three independent experiments. Medium without microorganisms was used as a negative control. Biofilm formation was measured as an optical density (OD_570_) and interpreted as either weak, moderate, or strong biofilm formers according to the criteria of Saxena et al. [33,35].

### 2.5. Mono- and Dual-Species Biofilm: In Vitro Dynamic Biofilm Model

Mono- and polymicrobial biofilms were also developed under dynamic shear conditions using the CDC biofilm reactor.

Biofilms were grown on polycarbonate, polypropylene, and Teflon coupons inserted into rods (three coupons per rod) mounted in a CDC biofilm reactor according to the recommendations of the U.S. Environmental Protection Agency.

Briefly, 400 mL TSB supplemented with 1% glucose was inoculated with 1 mL of an overnight culture of the microorganisms alone or together at 10^8^ CFU/mL concentration mixed at a 1:1 ratio. Each culture was incubated in the biofilm reactor with 125 ± 5 r/min stirring at 21 ± 2 °C for 24 h in batch mode.

After 24 h, a constant flow of 11.7 ± 0.2 mL/min of a total of 20 L medium for a 24/48 h of further incubation under continuous flow (continuous-flow stirred-tank reactor, CSTR), was applied following the ASTM Standards E2562-12 [36]. Biofilm accumulation developed on the surface of coupons was assessed as the total number of viable microorganisms. In fact, after 24 (to value biofilm formation) or 48 h (to value eradication of mature biofilm) coupons were aseptically removed from the chamber and planktonic cells were eliminated by rinsing with 1 mL sterile PBS.

For enumerating CFU of coupons, the biofilms were detached by scraping the coupon surface with a sterile wooden stick. Then, biofilm cells were suspended in 3 mL PBS and vortexed for 5 min. The aliquots were serially diluted, plated for CFU counting, and the cell densities in log_10_ CFU/cm^2^ of surfaces of the coupons were calculated.

The following formulae were used as the ASTM Standard: E2562-17 [36].

Log_10_ (CFU/cm^2^) = Log_10_ (mean CFU/volume plated) × (volume scraped/surface coupon) × (dilution)

### 2.6. Eradication of Established Biofilm

Treatment of static and dynamic biofilms was done with different concentrations ranging from 5 to 75 µg/mL of *Allium* sp. extracts. Mature biofilms were incubated for 24 h with our extracts, after which time they were collected and sonicated. Finally, the eradication effect of the extracts on the biofilm structures was compared with the control group measuring the minimum biofilm eradication concentration (MBEC) enumerating the CFUs.

*C. albicans* and *K. pneumoniae* were plated on TSB agar supplemented with chloramphenicol (Sigma-Aldrich, United States) for the selective growth of *C. albicans* or with amphotericin B (Sigma-Aldrich, United States) for the selective growth of *K. pneumoniae.*

Briefly, after incubation, the content of the wells/coupons was discarded and biofilms were serial diluted and counted as previous reported.

Moreover, for each treatment we calculated the percentage of killing following the formula:

% Kill = (1.10^-LR^)·100, (1)

LR = Mean Log_10_ Untreated Coupons − Mean Log_10_ Treated Coupons. (2)

The log_10_ density for each coupon is calculated as follows:

Log_10_ (CFU/coupon) = Log_10_ (X/B) × (V/D), (3)

where X = average CFU of the replicate sample plates, B = volume plated, V = volume of extracts D = 10^-k^, and k = dilution as reported in ASTM Standard: E2562-17 [36].

### 2.7. Statistical Analysis

Statistical analyses were performed using SigmaPlot 11.0 (Systat Software, Inc., USA).

Values are expressed as the mean ± standard deviation (±SD). Statistical comparisons between microbiological mean log values were performed using the one-way ANOVA (*p* < 0.001). Pairwise multiple comparison was applied to different compounds and selected materials (Holm–Sidak test, *p* < 0.05).

## 3. Results and Discussion

This is the first report on two different extracts from botanical products obtained with a rapid extraction method (the Naviglio extractor) and tested for their antibiofilm activity. The active process due to the high pressure inside and the low pressure outside permitted the extraction of substances from the solid matrix in a short period of time and at low temperature with a good reproducibility of the extraction and above all the production of high-quality extracts that are phyto-complex with a synergic action [28].

Both compounds were extracted from the leaves of two fresh species of *Allium*, and the action of their sulfur compounds [37] toward microorganisms was tested because it is known that the antimicrobial action of sulfur compounds depends on their hydrophilic or lipophilic character [38].

In this study, first we evaluated microbial attachment and biofilm formation on the different materials with various physicochemical properties.

Therefore, we used in parallel the static model of biofilms grown in microtiter plates previously experienced in our laboratory and a dynamic model of biofilms grown in the CDC reactor which allows the simulation of dynamic conditions during biofilm formation.

In each experiment, two microorganisms, *C. albicans* and *K. pneumoniae* alone or together, were compared since both can colonize different substrates (sand, plastic, stainless steel, skin surfaces, blood, liver, and other organs) in a large range of environmental conditions.

*C. albicans* and *K. pneumoniae* interactions modulate their local chemistry environment in multiple ways to create niches favorable to their growth and survival. These biofilm communities, formed in the host or on abiotic surfaces such as medical devices, can increase virulence, drug tolerance, and immune evasion [39].

The MIC and MBC/MFC of the *Allium* extracts were evaluated on both *K. pneumoniae* and *C. albicans* at concentrations ranging from 5 to 250 μg/mL and the results are presented in Table 1.

The two extracts showed an antimicrobial activity against both tested strains and the most susceptible was *C. albicans* with an MIC of 75 μg/mL and an MFC of 150 μg/mL for the *A. ursinum* extract, and 80 μg/mL MIC and 100 μg/mL MFC for the *A. oschaninii* extract.

**Figure 1:**
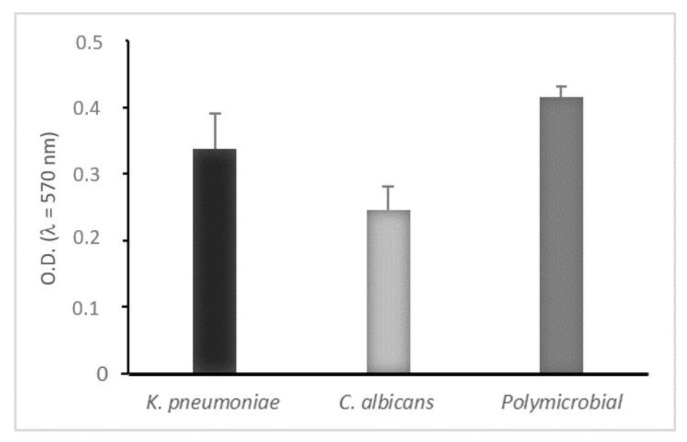
Biofilm biomass quantitation of mono- and polymicrobial biofilms formed on polystyrene microtiter plates (static conditions) after 24 h. Biofilm formation measured by crystal violet light absorbance at 570 nm (optical density, OD_570_). Negative (OD ≤ ODc), weakly adherent (ODc < OD ≤ 2 × ODc), moderately adherent (2 × ODc < OD ≤ 4 × ODc), strongly adherent (4 × ODc < OD). Results display the mean ± SD of three independent experiments.

Both microorganisms were able to form consistent monomicrobial and polymicrobial biofilms in static way and the crystal violet biofilm assay was used to quantify them, as shown in Figure 1.

We observed that *K. pneumoniae* was a strong biofilm producer while *C. albicans* showed moderate biofilm production, but when they formed a mixed biofilm the adherence and growth of both microorganisms were affected by the presence of each other and the result was a strong polymicrobial biofilm, corroborating the idea that, in polymicrobial biofilms, proteins secreted by microorganisms may play important roles in interactions [40,41].

**Figure 2:**
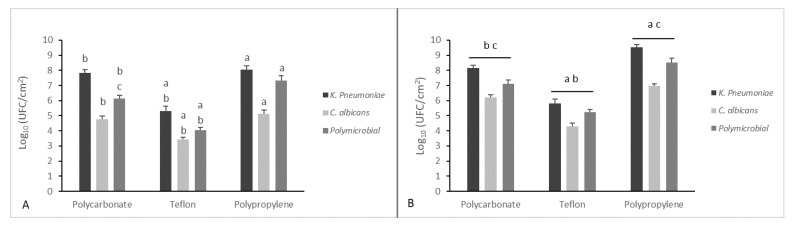
Quantification of viable cells of mono- and polymicrobial biofilms from a CDC biofilm reactor using three different coupon surfaces (panel **A**) and the same surfaces grown in a static way (panel **B**). Results display the mean ± SD (error bars) of three independent experiments. Statistical analysis was by one-way analysis of variance (ANOVA) with Holm–Sidak test. *p*-value < 0.001. Significant differences are reported with a = polypropylene vs. Teflon, b = polycarbonate vs. Teflon, and c = polypropylene vs. polycarbonate.

In Figure 2, total biofilms produced by *K. pneumoniae* and/or *C. albicans* on three different support materials, in a dynamic and static way, are quantified.

Teflon, polypropylene, and polycarbonate coupons were used in CDC biofilm reactor containing 400 mL of TSB that had been inoculated with an individual strain of *K. pneumoniae* and *C. albicans* to obtain 6 log CFU/mL populations. The reactor was set with a constant flow rate at 11.7 ± 0.2 mL/min for 24 h. Both the microorganisms demonstrated the capacity to form mono- and polymicrobial biofilms on all three coupons in a dynamic way showing an adhesion of up to 70%. There were differences between the three materials and the two microorganisms.

Results indicated that rougher textures (polypropylene) had more microbial attachment and biofilm formation than those with smoother textures (Teflon). The thickest biofilms were obtained on Teflon [42].

These materials differ in their physicochemical surface properties and surface texture, leading to different interactions with the microorganisms during initial attachment and expansion of the biomass. In dynamic conditions, the strain that is more strongly biofilm forming, on all the tested materials, remains *K. pneumoniae*, and in these conditions the polymicrobial biofilm is once again less strong than the latter. Differences between the three different materials and the two different microorganisms are significant except for polypropylene and polycarbonate in which biofilm formation was the same for both microorganisms.

We noticed a general increase of biofilm growth on coupons in static conditions compared to dynamic ones. Significant differences between the three materials were found when these were grown in a static way.

To assess the antibiofilm efficiency of the two extracts sub-MICs of them were selected for the study of biofilm eradication made in a static and dynamic way.

**Figure 3:**
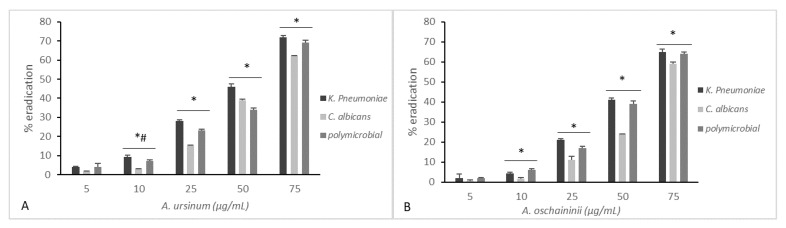
Comparison of minimal biofilm eradication concentration (MBEC) of the *A. ursinum* (panel **A**) and *A. oschaninii* (panel **B**) extracts on mono- and polymicrobial mature biofilms grown in static conditions in 96-well polystyrene plates. Testing performed in triplicate. Error bars represent standard deviation. Statistical analysis was by one-way analysis of variance (ANOVA) with Holm–Sidak test. Comparison of different treatment groups showed a significant reduction in log density for the two strains alone or together. (* *p* value < 0.001, ^#^ *p* value < 0.05 for 10 vs. 25).

**Figure 4:**
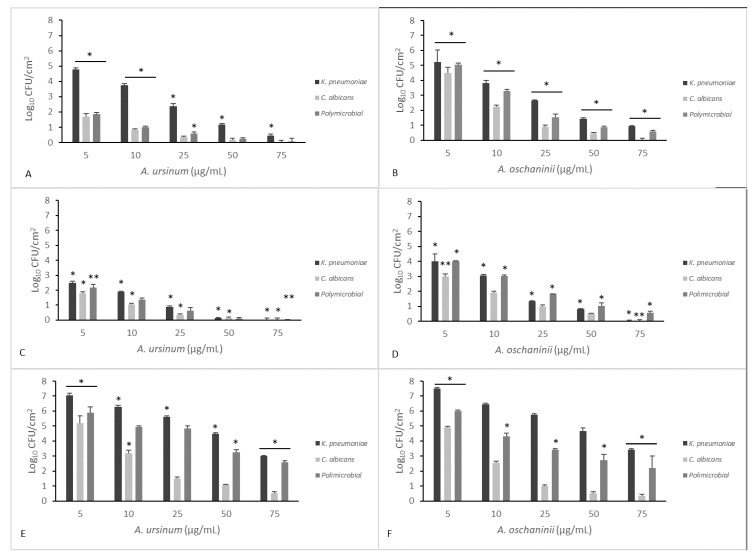
Comparison of minimal biofilm eradication concentration (MBEC) of the *A. ursinum* and *A. oschaninii* extracts on mono- and polymicrobial mature biofilms grown in dynamic conditions in a CDC reactor on different materials (**A**,**B** polycarbonate; **C**,**D** Teflon; **E**,**F** polypropylene). Testing performed in triplicate. Error bars represent standard deviation. Statistical analysis was by one-way analysis of variance (ANOVA) with Holm–Sidak test. Significance is valuated between different compound concentrations tested on the same coupon. (* *p* value < 0.001; ** *p* value < 0.05).

Figure 3 and Figure 4 summarize the effects of the two extracts on monoculture and mixed mature biofilms grown in static and dynamic manner.

After 48 h, wells/coupons were treated with both extracts at 5, 10, 25, 50, or 75 μg/mL for 24 h. Treatments at varying concentrations of the two *Allium* extracts on mono- and polymicrobial static biofilms are displayed in Figure 3. Both extracts showed significant reduction in biofilms in a dose-dependent manner even if we can observe that the *A. ursinum* extract (Figure 3A) showed the most effective and constant effects, while the eradication with *A. oschaninii* was more efficient only at major doses. The differences in the mean values among the treatment groups are greater than would be expected by chance; there is a statistically significant difference (*p* ≤ 0.001). All pairwise multiple comparison procedures (Holm–Sidak method) showed an overall significance level except for lower concentrations for both microorganisms alone or together.

In Figure 4, eradication treatments on mono- and polymicrobial CDC-biofilms are reported. Surviving populations were determined by scraping off the coupons and plating on selective media. Extracts at 50 μg/mL significantly inactivated all biofilms on both surfaces of Teflon and polycarbonate, compared with control (*p* < 0.05). A maximal reduction of 7 log_10_–1 log_10_ CFU/cm^2^ was obtained at the higher concentration used in the experiment (75 μg/mL).

To allow the parallel measurement of biofilm killing and biofilm removal eliminating any experiment-to-experiment variability, the CFU/coupons and percentage of killing were calculated. The percentage of killing used to evaluate the total microbicidal efficacy of the two *Allium* extracts showed a value of about 99% of microorganisms killed when we calculated the mean of different concentrations of *Allium* on the different materials together. In fact, the LR (Mean Log_10_ Untreated Coupons − Mean Log_10_ Treated Coupons) previously reported was included between 4 and 5.5. We obtained an increased cell removal when the surfaces were polycarbonate and Teflon, which anyway showed a weaker biofilm formation.

As previously described, the standardized reactors that have been used in medical research tend to mimic the built environment of biofilms, we showed that biofilms grown in the CDC reactor could be considered a valid replacement for miming all the clinical conditions in which biofilms could be found. Although a wide range of distinct biofilms could be found, we detected more complex and robust tridimensional structures than in microplates [27,43,44].

Since the intrinsic potency of the extracts against planktonic cultures (MIC values) is essentially of the same order of magnitude against the two strains, great variation in the activity of each individual extracts against the same strains when analyzed for their biofilm structure has been noticed.

The differences in the mean values among the treatment groups are greater than would be expected by chance; there is a statistically significant difference (*p* < 0.001).

Considering that, physical properties of support, including hydrophobicity, surface roughness, and electrostatic charges are important for the initial attachment step of bacteria and yeast [45], our results indicate that extracting some of the natural sulfur compounds through a rapid temperature control method to avoid the volatilization on extraction with the Naviglio’s method, exhibited promising eradication activities on mono- and polymicrobial biofilms, also when grown in CDC reactor, and they warrant more consideration as prospective antimicrobials.

Our results showed that Teflon has lower surface energies when compared with those of the two other materials and contains fewer irregularities that served as settlement sites which could explain the much lower level of colonization on the surface of this material. On the other hand, we could have an easier eradication with our compounds. In our study, we confirm the efficacy of our extracts even for mono- and polymicrobial biofilms grown in CDC reactor [46].

In hospital environments, multidrug-resistant and biofilm-producing bacteria are correlated to the difficulties associated with treating human infections and eradicating them, constituting a real public health problem [47].

*K. pneumoniae* and *C. albicans* are associated with healthcare-associated infections (HAI) [48].

Biofilms have been implicated in the development of many infections, including those related to medical devices [49,50].

Therefore, nowadays, studying different materials in vitro by reproducing the dynamic conditions and evaluating how biofilms are formed on them, seems to be actual and more important. Furthermore, focusing on the eradication biofilm capacity of some natural substances could be an advantage to counter drug-resistance phenomena and achieve good results even in hospitals settings and on medical devices to counter HAIs.

## Figures and Tables

**Table 1:** Minimum inhibitory concentration (MIC) and minimum bactericidal/fungicidal concentration (MBC/MFC) of *Allium ursinum* and *Allium oschaninii* extracts against test microorganisms.

	*A. ursinum*			*A. oschaninii*		
	MIC (µg/mL)	S.D.	MBC/MFC (µg/mL)	MIC (µg/mL)	S.D.	MBC/MFC (µg/mL)
***Klebsiella pneumoniae* ATCC 10031**	150	0.05	200	200	0.06	250
***Candida albicans* ATCC 90028**	75	0.07	150	80	0.03	100
